# The Association of Periodontal Status, Salivary Flow Rate, Salivary Cortisol Levels, and Cytokine Levels with Cognitive Status in Elderly Subjects

**DOI:** 10.3390/geriatrics10050127

**Published:** 2025-09-23

**Authors:** Mirsarinda Anandia Leander, Zalfa Karimah, Sandra Olivia Kuswandani, Robert Lessang, Sri Lelyati C. Masulili, Benso Sulijaya, Dimas Ilham Hutomo, Herlis Rahdewati, Koichi Tabeta, Fatimah Maria Tadjoedin

**Affiliations:** 1 Periodontics Specialist Program, Department of Periodontics, Faculty of Dentistry, Universitas Indonesia, Jakarta 10430, Indonesia; mirsaleander85@gmail.com (M.A.L.); sasa.karimah@gmail.com (Z.K.); 2 Department of Periodontics, Faculty of Dentistry, Universitas Indonesia, Jakarta 10430, Indonesia; sandra.olivia01@ui.ac.id (S.O.K.); robertlessang@gmail.com (R.L.); srilelyati@yahoo.com (S.L.C.M.); bensosulijaya@gmail.com (B.S.); dimas.hutomo@ui.ac.id (D.I.H.); herlis.rahdewati02@ui.ac.id (H.R.); 3 Graduate Research Program, UCL Eastman Dental Institute, Gower St., London WC1E 6AE, UK; 4 Division of Periodontology, Faculty of Dentistry & Graduate School of Medical and Dental Sciences, Niigata University, Niigata 951-8514, Japan; koichi@dent.niigata-u.ac.jp

**Keywords:** elderly, cognitive status, periodontal status, salivary flow rate, salivary cortisol levels, cytokine levels

## Abstract

**Background/objectives:** Aging is associated with a decline in physiological and cognitive functions. Periodontitis, a disease affecting the periodontal tissues, increases in prevalence with age. Bacteria and inflammatory mediators resulting from periodontitis can trigger neuroinflammation and potentially accelerate the progression of neurodegenerative diseases. This study aimed to evaluate the association between periodontal status, salivary flow rate, salivary cortisol levels, and cytokine levels with cognitive status in elderly Indonesian subjects. **Methods:** This cross-sectional study involved 70 participants aged ≥ 60 years from several social institutions in Jakarta and the Dental Hospital, Faculty of Dentistry, Universitas Indonesia. All participants provided written informed consent before the examination. Periodontal parameters, including plaque score, calculus index, bleeding on probing, number of remaining teeth, and functional tooth units, were assessed. Unstimulated salivary flow was collected over five minutes, and salivary cortisol levels were measured. Gingival crevicular fluid samples from the deepest periodontal pockets were collected to measure cytokine levels (TNF-α and IL-1β). Both cortisol and cytokine levels were analyzed using ELISA. Cognitive function was evaluated using the Hopkins Verbal Learning Test. **Results:** Plaque score, calculus index, and bleeding on probing were moderately associated with cognitive scores (*p* < 0.05). In contrast, the number of remaining teeth, functional tooth units, periodontitis severity, salivary flow rate, salivary cortisol, and cytokine levels were not significantly associated with cognitive scores (*p* > 0.05). **Conclusions:** These findings suggest that elderly individuals with cognitive impairment tend to have poorer periodontal health than those with normal cognitive function.

## 1. Introduction

According to the United Nations World Population Prospects 2024, the global population in 2024 is estimated at 8.2 billion, with individuals aged 65 years or older comprising an increasingly significant proportion [1]. Currently, the number of individuals aged 60 and above has increased from 1 billion in 2020 to 1.4 billion in 2024. It is estimated that at least one in six people globally will be aged 60 years or older in 2030 [2]. Based on demographic data in Indonesia, 12 percent of the population in 2024 was classified as elderly. The elderly population is projected to reach 65.82 million, or 20.31 percent of the total population, in 2045 [3]. Therefore, Indonesia is currently transitioning into an aging population. As people age, physiological functions decline due to degenerative processes, leading to an increased prevalence of non-communicable diseases such as dementia and periodontal disease. This trend is consistent with the findings of Steiner et al. [4].

Mild Cognitive Impairment (MCI) is characterized by cognitive decline greater than expected for normal aging but not severe enough to be classified as dementia [5]. Early identification of its risk factors is crucial for preventing the progression to dementia. Non-modifiable risk factors include age, sex, and genetic predisposition, while modifiable factors involve diet, lifestyle, and medical conditions such as hypertension, diabetes, and periodontitis [6].

Periodontitis is a chronic inflammatory disease of the tooth-supporting tissues with a high prevalence in Indonesia, ranging from 19.6% to 27.3% in 2022. Among the elderly, 19.3% experience oral health problems, 38.4% have cognitive impairment, and 17.3% suffer from depression [7]. The prevalence of periodontitis increases with age. Clinical periodontal parameters such as probing depth, clinical attachment loss, and alveolar bone loss in patients with periodontitis have been positively associated with cognitive decline [8]. The severity of periodontitis is also associated with a significant decrease in salivary flow rate [9]. Saliva has been proven to be very important for protecting teeth and periodontal tissue by maintaining the balance of the oral microbiota [10]. Oral bacteria are not only limited to causing local oral diseases like periodontitis but can also contribute to systemic conditions by triggering systemic inflammation. Emerging studies have shown that the oral microbiome may play a role in the onset of neuroinflammation in neurodegenerative diseases [11,12].

Several studies have investigated the association between periodontitis and cognitive impairment, including dementia and Alzheimer’s disease. Studies examining the relationship between periodontitis and cognitive function have shown varying results. Research by Tadjoedin et al. demonstrated an association between periodontitis and cognitive impairment in the elderly, based on periodontal status and subgingival microbiota [13]. In contrast, a systematic review by Tonsekar et al. noted that the evidence from existing literature regarding chronic periodontitis and extensive tooth loss as risk factors for dementia remains inconclusive [14].

A systematic review by Nascimento et al. concluded that individuals with periodontitis have a higher risk of developing cognitive impairment. This may be due to bacteria from dental plaque entering the bloodstream, where pathogenic products can cross the blood–brain barrier and reach the brain. Another possible mechanism involves periodontal pathogenic bacteria initiating increased serum levels of inflammatory mediators such as IL-6, TNF-α, and CRP [15]. Another study by Ide et al. found that patients with Alzheimer’s disease had poor oral hygiene, and the presence of periodontitis was associated with greater cognitive decline. The study also examined systemic inflammatory markers, including CRP, TNF-α, and IL-10, and reported increased levels of pro-inflammatory cytokines and decreased levels of anti-inflammatory cytokines. However, the associations between these inflammatory markers and cognitive status were not statistically significant [16]. Marzabadi et al. conducted a study to assess the correlation between cytokine levels (TNF-α, IL-1β, and IL-6) and Alzheimer’s disease, which was categorized into three groups: mild, mild-to-moderate, and moderate-to-severe. This study reported that levels of TNF-α and IL-6 were higher in patients in the moderate to severe group, showing a negative correlation with cognitive function. However, the differences in IL-1β levels between groups were not statistically significant [17].

Patients with periodontitis often experience a decline in masticatory function due to tooth mobility and even tooth loss, which can lead to reduced salivary flow rate [18]. A key index used to evaluate masticatory function and performance is the number of functional tooth units (FTUs) [19]. A study by Ueno et al. reported that having ≥20 natural teeth and ≥8 FTUs was associated with reduced difficulty in chewing [18].

Salivary secretion is influenced by various factors. A study by Do et al. concluded that salivary flow rate is independently associated with cognitive impairment among the elderly in Korea [20]. Collins et al. further suggested that degeneration of the central nervous system in cognitive impairment may alter afferent or efferent reflexes, thereby reducing the salivary flow rate [21].

Other studies on saliva have shown that cognitive function can be assessed through salivary cortisol levels. Cortisol is a hormone produced and secreted by the adrenal cortex that regulates various brain functions such as metabolism, blood glucose levels, immune response, anti-inflammatory actions, blood pressure, emotional regulation or stress response, and overall mood [22]. Saliva reflects the overall condition of the human body and is therefore widely used in research due to its ease of collection, storage, efficiency, and straightforward analysis. It serves as a diagnostic tool for systemic diseases and can be used as a parameter to monitor disease progression or recurrence [23].

Previous studies have indicated a relationship between periodontitis and cognitive impairment; however, some research has reported no significant association between the two. Data on the role of pro-inflammatory cytokines in cognitive impairment among patients with periodontitis, as well as the periodontal status based on clinical parameters in individuals with cognitive decline, remain limited. A decrease in salivary flow rate in older adults may alter the oral microbiota, thereby increasing the risk of periodontitis and potentially affecting cognitive function. Additionally, imbalanced salivary cortisol levels may contribute to changes in cognitive function among the elderly. Nevertheless, the roles of salivary flow rate and salivary cortisol in cognitive status in older adults remain underexplored, particularly in Indonesia. Therefore, this study aims to evaluate the association between periodontitis and cognitive impairment in the elderly by assessing periodontal status, pro-inflammatory cytokines, salivary flow rate, and salivary cortisol levels, which may contribute to an increased risk of cognitive impairment.

## 2. Materials and Methods

### 2.1. Ethical Clearance

This study received ethical approval from the Research Ethics Committee of the Faculty of Dentistry, Universitas Indonesia, under approval letter number 20/Ethical Approval/FKGUI/III/2024.

### 2.2. Subjects

This study included elderly male and female subjects aged 60 years or older, who were categorized into two groups based on their cognitive status. The inclusion criteria were (1) being diagnosed with periodontitis based on the 2017 World Workshop classification and (2) normal hearing ability. Exclusion criteria included (1) history of systemic diseases such as hypertension, diabetes mellitus, heart disease, blood disorders, or cancer; (2) current use of systemic medications (e.g., anti-hypertensives, anti-inflammatories, diuretics, antidepressants, antihistamines); (3) antibiotic use within the past three months; (4) history of radiation therapy or brain injury; (5) regular alcohol consumption; (6) smoking; (7) use of removable dentures; (8) complete edentulism; and (9) periodontal treatment within the past six months. Written informed consent was provided to all subjects. We estimated a total sample size of 64 participants (32 per group) using G*Power version 3.1.9.4, based on parameters from a previous study by Hategan et al., which included periodontal and cognitive scores [24].

### 2.3. Examination of Clinical Measurements

**Figure 1:**
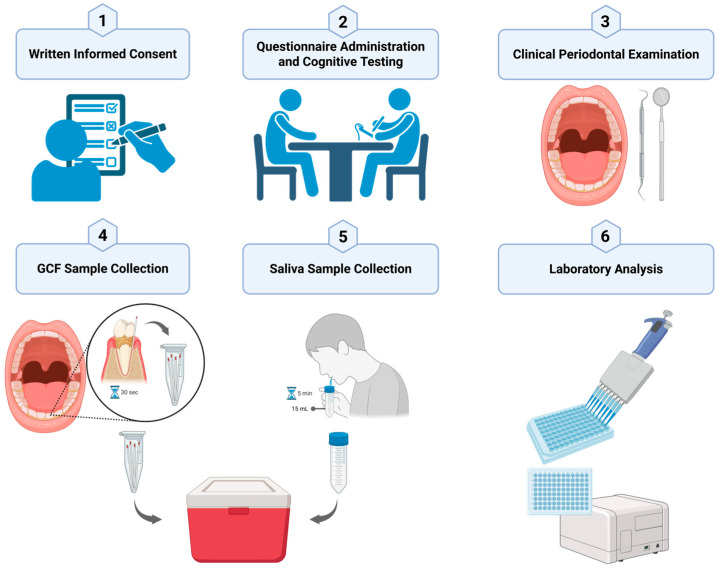
Research Workflow: (1) After selecting subjects who met the inclusion criteria, written informed consent was obtained; (2) Interviews were conducted to complete questionnaires and assess cognitive function using the HVLT instrument; (3) Clinical periodontal parameters were examined, including plaque score, bleeding on probing, calculus index, number of teeth, functional tooth units, and severity of periodontitis; (4) GCF samples were collected by inserting paper points into the deepest periodontal pockets; (5) Unstimulated saliva samples were collected on a separate day in the morning, using 15 mL sterile tubes. Both samples were immediately placed in a cooler box and transported to the laboratory for further analysis; (6) laboratory analysis was performed using the ELISA method to measure the levels of pro-inflammatory cytokines IL-1β and TNF-α in the GCF, as well as cortisol levels in the saliva. Created in BioRender. Kuswandani, S. (2025) https://BioRender.com/isq35vh.

This study conducted a calibration process by two examiners (M.A.L. and Z.K.) prior to sample collection at the study sites, resulting in an intraclass correlation coefficient (ICC) of 0.842 and a Kappa value of 0.800. The recruitment of research subjects who met the inclusion and exclusion criteria was conducted through interviews and the completion of questionnaires. This process included obtaining informed consent, recording personal data, conducting a general health examination, a clinical periodontal examination, and a cognitive assessment, as summarized in the workflow presented in Figure 1.

A clinical periodontal examination was performed using a UNC-15 probe, which included the assessment of

- The plaque score developed by O’Leary, Drake, and Naylor was used. This index evaluates the amount of plaque present on tooth surfaces. For each tooth, six sites are evaluated, comprising the mesiobuccal, mid-buccal, distobuccal, mesiolingual, mid-lingual, and distolingual surfaces. To determine the score of plaque-covered surfaces, first calculate the total number of available tooth surfaces by multiplying the number of present teeth by six (the number of examined sites per tooth). Then, count the number of surfaces with visible plaque and multiply this value by 100. Finally, divide the result by the total number of surfaces to obtain the proportion of plaque-positive sites expressed as a percentage [25,26].
- Bleeding on probing was calculated based on the method described by Lang et al., using six sites per tooth, comprising the mesiobuccal, mid-buccal, distobuccal, mesiolingual, mid-lingual, and distolingual surfaces. The calculation of the bleeding on probing followed the same approach as that used for plaque scoring [25,27].
- The calculus index developed by Ramfjord was used. This index evaluates calculus accumulation at two sites per tooth—buccal and palatal/lingual surfaces—by assessing both supragingival and subgingival deposits. A calculus score was recorded for each surface, then summed and divided by the number of teeth examined. The scoring criteria were as follows: 0—absence of calculus; 1—supragingival calculus extending slightly below the free gingival margin; 2—moderate amounts of supragingival and/or subgingival calculus; 3—heavy accumulation of both supragingival and subgingival calculus [25,28].
- Probing pocket depth (PPD) and clinical attachment loss (CAL) were measured using a periodontal probe to determine the staging of periodontitis. For each tooth, six sites are evaluated, comprising the mesiobuccal, mid-buccal, distobuccal, mesiolingual, mid-lingual, and distolingual surfaces [25]. PPD refers to the measurement from the free gingival margin to the base of the periodontal pocket, whereas CAL refers to the measurement from the cementoenamel junction (CEJ) to the base of the pocket [29].
- The number of remaining teeth and functional tooth units (FTUs) is used to evaluate masticatory performance. The number of remaining teeth was determined by counting all teeth present in the oral cavity, excluding third molars and retained roots from the calculation [30]. FTUs represent the number of opposing posterior tooth pairs—either natural or prosthetic—that are in contact. In this index, each contacting molar pair accounts for two units, while each premolar pair accounts for one unit, with a maximum possible score of 12 units [19].

Cognitive assessment was conducted using the Hopkins Verbal Learning Test (HVLT). Hogervorst et al. have validated the HVLT for dementia screening in Indonesia. Research by Hogervorst et al. indicates that a cut-off score of less than 14.5 on the HVLT should be used when screening for cognitive impairment [31]. In line with these criteria, subjects were categorized as cognitively impaired if their HVLT score was ≤14 and as cognitively normal if they scored between 15 and 36. The score obtained from the HVLT serves only as an indicator of cognitive impairment and is not designed for diagnostic purposes [32].

### 2.4. Saliva Sample Collection

Unstimulated saliva was collected by instructing subjects to passively drool into a tube over five minutes. Saliva collection was conducted between 5:30 a.m. and 6:30 a.m., before breakfast. While seated, subjects were instructed to swallow once before the collection began. The saliva was collected in 15 mL tubes and immediately placed in a cooler. After all samples were obtained, the salivary flow rate was calculated by dividing the total volume by five minutes. Cortisol concentrations in saliva were analyzed using the Enzyme-Linked Immunosorbent Assay (ELISA) method.

### 2.5. GCF Sample Collection

Gingival crevicular fluid was collected to measure cytokine levels of IL-1β and TNF-α using sterile paper points, which were carefully inserted into the deepest periodontal pocket for 30 s to avoid gingival bleeding. Samples contaminated with blood or saliva were excluded. The paper points were then placed into Eppendorf tubes containing 200 µL of PBS and stored at −20 °C. Subsequently, the samples were examined using the ELISA method.

### 2.6. Statistical Analysis

After collecting all the data, we processed and analyzed it using SPSS version 27 software. The study’s analysis encompassed both univariate and bivariate methods. Univariate analysis was conducted to characterize the individual variables, while bivariate analysis focused on exploring the differences or relationships between two variables. This bivariate analysis involved comparative tests (independent t-test, Mann–Whitney test, and chi-square test) and correlational tests (Pearson and Spearman). A *p*-value of <0.05 was considered statistically significant.

## 3. Results

Samples were collected from several social institutions in Jakarta and the Dental Hospital, Faculty of Dentistry, Universitas Indonesia, comprising a total of 70 subjects. Clinical periodontal examination, salivary flow rate, salivary cortisol levels, cytokine levels from GCF, and cognitive status were assessed and statistically analyzed to compare and correlate findings between groups.

### 3.1. Characteristics of Subjects

Table 1 presents data from 70 subjects, 49 of whom had cognitive impairment, ≤9 years of education, fewer than 20 teeth, 0–4 functional tooth units, and stage IV periodontitis. A statistically significant difference was observed in the education variable between subjects with normal cognition and those with cognitive impairment (*p* < 0.05), whereas the other variables (age, number of teeth, functional tooth units, and severity of periodontitis) showed no significant differences (*p* > 0.05).

### 3.2. Comparisons of Periodontal Status, Salivary Flow Rate, Salivary Cortisol Levels, and Pro-Inflammatory Cytokine Levels in Subjects with Normal Cognition and Cognitive Impairment

The comparisons of periodontal status, salivary flow rate, salivary cortisol levels, and pro-inflammatory cytokine levels in elderly subjects with normal cognition and cognitive impairment are shown in Table 2. There were statistically significant differences (*p* < 0.05) in periodontal status (plaque score, calculus index, and bleeding on probing) between subjects with cognitive impairment and those with normal cognition, whereas no significant differences were found in salivary flow rate, salivary cortisol levels, or cytokine levels. However, subjects with normal cognition tended to have higher mean salivary flow rates compared with those with cognitive impairment. Moreover, subjects with cognitive impairment exhibited higher cytokine levels (both IL-1β and TNF-α) compared with subjects with normal cognition.

### 3.3. Cognitive Score Correlations to Sociodemographic Status, Periodontal Status, Salivary Flow Rate, Salivary Cortisol Levels, and Pro-Inflammatory Cytokine Levels

The correlation analysis demonstrated a significant relationship (*p* < 0.05) between cognitive score and periodontal status, comprising plaque score, bleeding on probing, and calculus index. The correlation coefficients indicated a moderate negative relationship: plaque score (r = −0.289), calculus index (r = −0.279), and bleeding on probing (r = −0.335). This indicates that higher levels of plaque, calculus, and gingival bleeding are associated with lower cognitive scores (i.e., greater cognitive impairment). Meanwhile, the number of residual teeth and functional dental units, severity of periodontitis, levels of proinflammatory cytokines IL-1β and TNF-α, salivary flow rate, and salivary cortisol levels have no significant correlation with cognitive score (Table 3).

## 4. Discussion

The results of this study showed that educational level is one of the risk factors influencing cognitive impairment. Based on the analysis of years of education and cognitive status, there was a statistically significant difference (*p* = 0.002) between subjects with normal cognition and those with cognitive impairment. The relationship was also found to be negative and statistically significant (*p* < 0.001, r = −0.385). This finding aligns with the study by Han et al., who reported that individuals with higher educational attainment are better equipped to maintain cognitive function and performance (*p* < 0.001) [33].

The cognitively impaired group showed higher mean and median periodontal parameters, including plaque score, calculus index, and bleeding on probing, with statistically significant differences and correlations (*p* < 0.05) for each variable. These findings align with Tadjoedin et al., who also reported significant differences and correlations between cognitive status and several periodontal indicators, including plaque index, oral hygiene index, papillary bleeding index, pocket depth, gingival recession, attachment loss, and tooth loss [34].

Comparative and correlation analyses of tooth count showed no significant difference or correlation between cognitively normal and impaired subjects, although most individuals in both groups had fewer than 20 teeth. Similarly, Zhang et al. found that most individuals with cognitive dysfunction had fewer than 20 teeth, although statistical significance between groups was not specified [35].

The number of functional tooth units (FTUs) did not differ significantly between cognitively normal and impaired subjects, nor was there a significant correlation. However, the majority of cognitively impaired individuals had only 0–4 FTUs. A systematic review by Galindo-Moreno et al. reported that tooth loss is associated with decreased cognitive function [36]. However, in this study, we did not present any directional analysis between missing teeth and periodontal status. Some studies mentioned that cognitive decline appears more closely linked to the loss of masticatory function rather than to the number of missing teeth. Poor chewing ability is associated with reduced nutrient intake, increasing the risk of malnutrition and cognitive decline [35,36,37]. Additionally, tooth loss in the elderly has been associated with limited oral health knowledge [38].

Comparative and correlation analyses showed no significant differences in periodontitis severity between cognitively normal and impaired subjects, possibly due to data distribution and the high prevalence of severe periodontitis (Stage III–IV) among participants. Similarly, Gu et al. reported no significant association (*p* > 0.05) [39], while Iwasaki et al. found a significant association between severe periodontitis and future cognitive decline in community-dwelling elderly individuals in Japan [40].

Proinflammatory cytokine levels (IL-1β and TNF-α) were not statistically different between two groups, though mean levels tended to be higher in the impaired group. Gil-Montoya et al. analyzed 29 serum inflammatory biomarkers to assess the impact of systemic inflammation due to periodontitis on cognitive impairment. The results showed no significant differences in IL-1β and TNF-α levels in mild to moderate periodontitis, although mean cytokine levels were higher in the impaired group. In severe periodontitis, TNF-α levels differed significantly between groups, while IL-1β did not, with mean cytokine levels lower in the impaired group. Thus, the study could not confirm that systemic inflammation associated with periodontitis plays a significant role in the etiology of cognitive impairment [41]. Elevated circulating levels of IL-6, IL-1, TNF-α, and CRP in the elderly are strongly associated with a higher risk of morbidity and mortality, with TNF-α and IL-6 serving as markers of frailty [42,43]. Hammami et al. investigated proinflammatory cytokines in frail elderly individuals, most of whom resided in nursing homes. Their findings showed a strong association between frailty and cognitive impairment. Frailty was also linked to elevated inflammation, as frail subjects had higher serum levels of CRP, TNF-α, and IL-8 compared to healthy individuals [44].

The results of this study demonstrated no significant differences in proinflammatory cytokine levels between elderly subjects with normal cognition and those with cognitive impairment. This outcome may be attributed to factors such as an unequal number of subjects between groups and the fact that most elderly participants resided in nursing homes, sharing similar characteristics and exhibiting signs of frailty, which are associated with increased systemic inflammation.

The results of the bivariate analysis in this study indicated that the mean salivary flow rate was higher in cognitively normal subjects compared to those with cognitive impairment. This finding is consistent with the study by Do et al., which demonstrated that the rate of unstimulated salivary flow significantly influences cognitive function. Specifically, unstimulated salivary flow was higher in elderly individuals with normal cognition compared to those with cognitive impairment [20]. Conversely, degeneration of the central nervous system in individuals with cognitive impairment may disrupt afferent or efferent reflexes, leading to a reduction in salivary flow rate [21].

In this study, unstimulated saliva was collected from subjects before they had breakfast, and the saliva collection was conducted between 05:30 and 06:30 a.m. Participants were seated and instructed to swallow once before the measurement began. They then continuously expectorated into a provided tube for five minutes. The results showed that 32 samples had unstimulated salivary flow rates below the normal threshold (<0.3 mL/min), although no statistically significant differences or correlations were observed.

Cognitive function can also be assessed by measuring salivary cortisol levels. Cortisol, which is secreted at the end of the hypothalamic–pituitary–adrenal (HPA) axis, significantly affects overall cognitive function. Higher cortisol levels are significantly associated with lower cognitive performance [22]. In this study, the mean salivary cortisol levels were higher in cognitively impaired subjects compared to cognitively normal subjects; however, the results of both the comparison and correlation tests did not show statistically significant differences or associations between salivary cortisol levels and cognitive scores. This lack of significance may be because salivary cortisol levels are strongly influenced by various external and internal factors, including physical activity, mood, depressive symptoms, and environmental changes throughout the day [45].

There is substantial scientific evidence and numerous research studies evaluating the relationship between periodontitis and cognitive decline. Emerging studies have also explored associations between periodontitis, salivary flow rate, cytokine and cortisol levels, and cognitive function. Therefore, accurate diagnosis and proper management of chronic periodontitis are essential for improving quality of life, especially in the elderly [15].

Managing highly complex geriatric patients requires Comprehensive Geriatric Health Services, including preventive, promotive, curative, rehabilitative, and palliative care, delivered holistically by an integrated team starting from the family and community levels. Acharya et al. identified several barriers to oral health care in Southeast Asia, including Indonesia—such as poor access to oral health services, uneven distribution of dental professionals, weak health information systems, private sector dominance, and insufficient or non-existent financing mechanisms for outpatient dental procedures [46].

To enhance the oral health of elderly individuals in Indonesia, several key solutions have been identified. These include educating caregivers and family members about oral health, expanding social security coverage, and fostering collaboration across various sectors. Teledentistry can improve access to vital information and provide timely recommendations for addressing oral health issues. In institutional settings, targeted interventions such as regular visits from dental professionals, equipping dental units with essential tools, and facilitating access to external dental services can prove beneficial.

This study has several limitations. First, a cross-sectional research design hinders the ability to establish causal relationships (cause and effect). Second, sampling was primarily conducted in a limited number of social care homes, where subjects shared similar characteristics, potentially limiting the generalizability of the findings. Nevertheless, the results of this study offer valuable insights into the connections between periodontal health, salivary flow rate, salivary cortisol levels, and cytokine levels with cognitive status in elderly individuals in Indonesia.

## 5. Conclusions

Elderly individuals with cognitive impairment tend to have poorer periodontal health and greater difficulty maintaining oral hygiene. This leads to increased plaque and calculus accumulation, as well as heightened gingival inflammation, when compared to cognitively normal individuals. Further research is needed to explore the relationship between salivary flow rate, salivary cortisol levels, and cytokine levels with cognitive status in elderly individuals in Indonesia.

## Figures and Tables

**Table 1:** Characteristics of subjects (N = 70).

Characteristics	Cognitive Status (N = 70)		*p*-Value
	Normal Cognition (n = 21)	Cognitive Impairment (n = 49)	
	n (%)	n (%)	
**Sociodemographic Status**			
Age			
60–69 years old	15 (71.4)	25 (51)	0.188
≥70 years old	6 (28.6)	24 (49)	
Education			
>9 years	15 (71.4)	14 (28.6)	0.002 *
≤9 years	6 (28.6)	35 (71.4)	
**Periodontal Status**			
Number of teeth			
≥20	11 (52.4)	20 (40.8)	0.529
<20	10 (47.6)	29 (59.2)	
Functional tooth unit			
≥5	5 (23.8)	9 (18.4)	0.845
0–4	16 (76.2)	40 (81.6)	
Severity of periodontitis			
Stage I–II	4 (19)	3 (6.1)	0.224
Stage III–IV	17 (81)	46 (93.9)	

Chi-square Test; * *p* < 0.05 = significantly different.

**Table 2:** Comparisons of periodontal status, salivary flow rate, salivary cortisol levels, and pro-inflammatory cytokine levels in elderly subjects with normal cognition and cognitive impairment (N = 70).

Variables	Cognitive Status (N = 70)				*p*-Value
	Normal Cognition (n = 21)		Cognitive Impairment (n = 49)		
	Mean ± SD	Median (Min–Max)	Mean ± SD	Median (Min–Max)	
**Periodontal Status**					
Plaque score (%)	63.8 ± 22.1	56 (17.7–93)	76.5 ± 18.9	83.3 (27–100)	0.028 ^b^*
Bleeding on probing (%)	32.9 ± 15.1	28.4 (10.4–59)	49.1 ± 22	50 (5.9–87)	0.003 ^a^*
Calculus index	1.1 ± 0.5	1 (0.3–2.1)	1.4 ± 0.5	1.6 (0.3–2.5)	0.017 ^a^*
**Salivary Flow Rate** (mL/min)	0.5 ± 0.4	0.4 (0–1.9)	0.4 ± 0.4	0.3 (0–1.6)	0.232 ^b^
**Salivary Cortisol Levels** (ng/mL)	0.1 ± 0.1	0 (0–0.7)	0.1 ± 0.3	0 (0–2.6)	0.568 ^b^
**Pro-inflammatory Cytokine Levels**					
IL-1β (pg/mL)	633.5 ± 536.8	467.2 (95.5–2710.5)	693.2 ± 630	554.35 (107–3073)	0.863 ^b^
TNF-α (pg/mL)	132.8 ± 93.9	118.4 (23–477.4)	137.4 ± 120.4	115.4 (26.3–653.5)	0.613 ^b^

^a^ = Independent T-Test; ^b^ = Mann–Whitney Test; * *p* < 0.05 = significantly different.

**Table 3:** Cognitive score correlations to sociodemographic status, periodontal status, salivary flow rate, salivary cortisol levels, and pro-inflammatory cytokine levels in elderly subjects (N = 70).

Cognitive Score (N = 70)	r	*p*-Value
**Sociodemographic Status**		
Age	−0.163	0.177 ^a^
Education	−0.385	<0.001 ^b^*
**Periodontal Status**		
Plaque score (%)	−0.289	0.015 ^b^*
Bleeding on probing (%)	−0.335	0.005 ^a^*
Calculus index	−0.279	0.020 ^a^*
Number of teeth	−0.014	0.907 ^b^
Functional tooth unit	0.041	0.738 ^b^
Severity of periodontitis	−0.176	0.145 ^b^
**Salivary Flow Rate** (mL/min)	0.212	0.078 ^b^
**Salivary Cortisol Levels** (ng/mL)	−0.103	0.396 ^b^
**Pro-inflammatory Cytokine Levels** (pg/mL)		
IL-1β	−0.145	0.230 ^b^
TNF-α	−0.106	0.381 ^b^

^a^ = Pearson Test; ^b^ = Spearman Test; r = correlation coefficient; * p < 0.05 = significant correlate.

## Data Availability

The data that support the findings of this study are available from the corresponding author (F.M.T.) upon reasonable request.
